# A proposal for a new classification of pes anserinus morphology

**DOI:** 10.1007/s00167-018-5318-3

**Published:** 2018-12-10

**Authors:** Łukasz Olewnik, Bartosz Gonera, Michał Podgórski, Michał Polguj, Hubert Jezierski, Mirosław Topol

**Affiliations:** 1 Department of Normal and Clinical Anatomy, Interfaculty Chair of Anatomy and Histology, Medical University of Lodz, Lodz, Poland; 2 Department of Angiology, Interfaculty Chair of Anatomy and Histology, Medical University of Lodz, Łódź, Poland; 3 Department of Trauma and Orthopaedic Surgery, Hospital of Ministry of Interior and Administration, Lodz, ul. Północna 42, 91-425 Łódź, Poland; 4 Polish Mother’s Memorial Hospital Research Institute, Lodz, Poland

**Keywords:** Gracilis tendon, New classification, Pes anserinus, Semitendinosus tendon

## Abstract

**Purpose:**

The pes anserinus (PA) is characterized by high morphological diversity. As the semitendinosus and gracilis muscle tendons are routinely harvested for the reconstruction of other tendons, especially the anterior cruciate ligament (ACL), it is of clinical importance. The presence of accessory bands within PA tendons can handicap the harvesting process. Therefore, the purpose of the study was to suggest a new morphological classification of the PA morphology.

**Methods:**

Classical anatomical dissection was performed on 102 lower limbs (56 right, 46 left) fixed in 10% formalin solution. The morphology and insertion of the PA (including accessory bands) were assessed, and morphometric measurements were taken.

**Results:**

In all cases, the PA was present and composed of the sartorius, gracilis and semitendinosus tendons. Six types of PA were distinguished based on the presence of accessory bands. The most common composed of monotendinous sartorius, gracilis and semitendinosus—54 limbs (52.9%). Additionally, three types of insertion were noted (short, band-shaped and fan-shaped). The mean length between the insertion and the origin of the accessory bands to the fascia of the gastrocnemius muscle was 63.5 mm.

**Conclusion:**

The morphology of the PA was highly variable. The gracilis and semitendinosus tendons often had accessory bands that would complicate the harvesting process. The planning of surgical procedures may be improved by our proposed classification.

**Electronic supplementary material:**

The online version of this article (10.1007/s00167-018-5318-3) contains supplementary material, which is available to authorized users.

## Introduction

The pes anserinus (PA) is anatomically defined as the conjoined tendons of three muscles that insert onto the anteromedial surface of the proximal part of the tibia. It is composed of the sartorius tendon (ST), gracilis tendon (GT) and the semitendinosus tendon (STT). In their distal part, near the insertion, the *Anserinus plate* is formed by the fusion of the tendons with the fascia of the leg. This tendon plate consists of superficial and deep layers, which both have an insertion on the medial side of the tibial tuberosity. The superficial layer is formed by the sartorius tendon, while the deep one is formed by the gracilis and semitendinosus tendons.

Previous studies have discussed the variability of the muscles and tendons present in the lower limb; however, the degree of variation in the PA is of major clinical importance due to its role as a source of grafts [1, 2, 9, 10, 14, 16, 17]. The most common variation lies with the accessory tendinous band, departing from the gracilis and/or semitendinosus tendon [1, 2, 9, 14, 16]. Their number varies individually [7, 14]. During embryological development, adhesions or accessory bands may occur between anatomical structures such as the tibial collateral ligament, superficial fascia, gastrocnemius aponeurosis and surrounding connective tissue layers [7, 14].

A strong autograft can be created with hamstring tendons. The semitendinosus and gracilis, in particular, are commonly grafted for the reconstruction of the anterior cruciate ligament (ACL) [1, 2, 9, 14, 16], medial knee reinforcement [1, 9], reconstruction of the patellar retinaculum after patellar subluxation, and for repairs to the patellar tendon after rupture [1]. Nevertheless, complications associated with PA tendon grafting have been reported, with the most common being tendon rupture [8]. In our opinion, one of the causes may be anatomical variations in this region.

Previous studies have described only variations, including the presence of accessory bands within the semitendinosus tendon [2, 9, 14, 16]. Accessory tendinous bands can be present in all PA tendons is our hypothesis. The aim of the study is to characterize variations in the morphology of PA tendons and their accessory bands. Second, to create a classification that can assist during the planning procedure of tendon grafting, particularly, adjusting a grafting technique to different types of the PA.

## Materials and methods

One hundred and two (56 right, 46 left) lower limbs fixed in 10% formalin solution were obtained from adult Caucasian cadavers. The median age of the cadavers was 61 years (35–88).

**Fig. 1:**
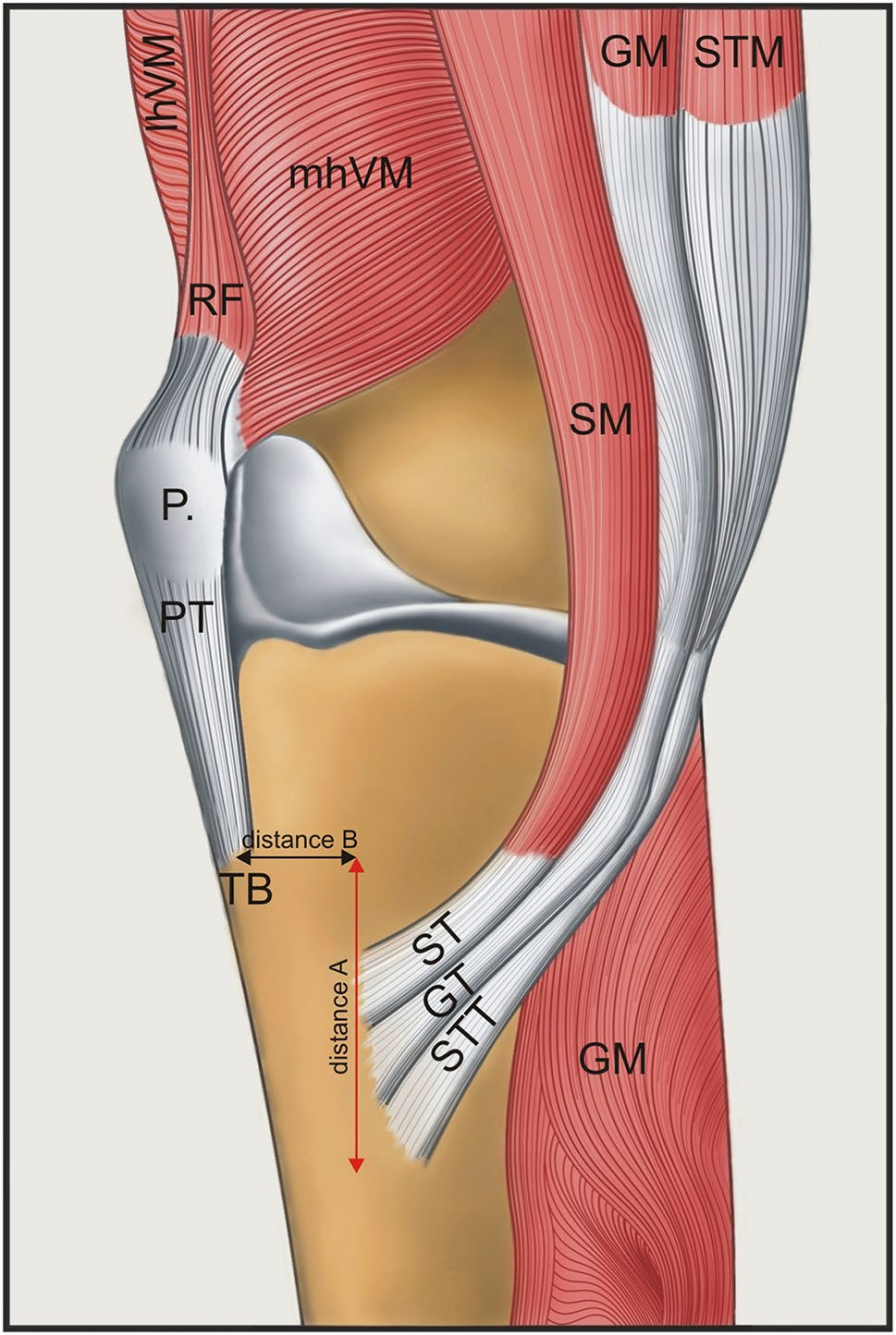
Schematic drawing of the pes anserinus and the potential line between tibial tuberosity and insertion of the pes anserinus. Red line indicates distance A—distally to the tibial tuberosity. Black line indicates distance B—medially to the tibial tuberosity. *lhVL* lateral head of the vastus lateralis, *mhVM* medial head of the vastus medialis, *RF* rectus femoris muscle, *P* patella, *PT* patellar tendon, *TB* tibial tuberosity, *SM* sartorius muscle, *ST* semitendinosus tendon, *GM* gracilis muscle, *GT* gracilis tendon, *STTM, STTM* semitendinosus muscle, *STT* semitendinosus tendon, *GM* gastrocnemius muscle

A dissection of the thigh and the crural region was performed using traditional techniques according to a strictly specified protocol [10–12]. Upon dissection, the following morphological features of the PA were evaluated:

- Types of PA morphology—including number of bands creating the PA.
- Types of the PA insertion (short, fan-shaped or band-shaped) and their location. To better characterize the exact location, measurements were taken of the inferior distance (line A) and medial distance (line B) to where the tibial tuberosity attaches to the structure (Fig. 1).
- The morphometric characteristics of tendon bands, such as their length and thickness, were measured, as were the crus length and distance between the insertion of the PA and the tibial tuberosity, the distance between the insertion of the PA and the origin of the accessory bands.

The first two sets of features were used to create a classification of the PA. They were chosen as the most important for the grafting procedure, particularly using a tendon stripper.

An electronic digital caliper was used for all measurements (Mitutoyo Corporation, Kawasaki-shi, Kanagawa, Japan). Each measurement was carried out twice with an accuracy of up to 0.1 mm.

The study procedure was approved by the Medical University of Lodz Bioethical Commission (Agreement No. RNN/297/17/KE).

### Statistical analysis

The statistical analysis was performed using Statistica 12 software (StatSoft Polska, Cracow, Poland). A *p* value below 0.05 was considered significant. The results are presented as mean and standard deviation, as well as the smallest and largest values, unless otherwise stated. The Chi^2^ test was used to compare the difference in the morphology of the PA between sexes and body sides.

Continuous data were checked for normality with the Shapiro–Wilk test. As the data was found to be not normally distributed, the Mann–Whitney test was used to compare the anthropometric and morphometric measurements between two muscles or two types of attachment. In case of multiple comparisons, the Bonferroni correction was applied. In the tables, the values underlined in red indicate significant differences.

## Results

### Variation in morphology of PA components

In 102 lower limbs (46 left and 56 right) the PA was dissected. Forty limbs were derived from females and 62 from males.

**Fig. 2:**
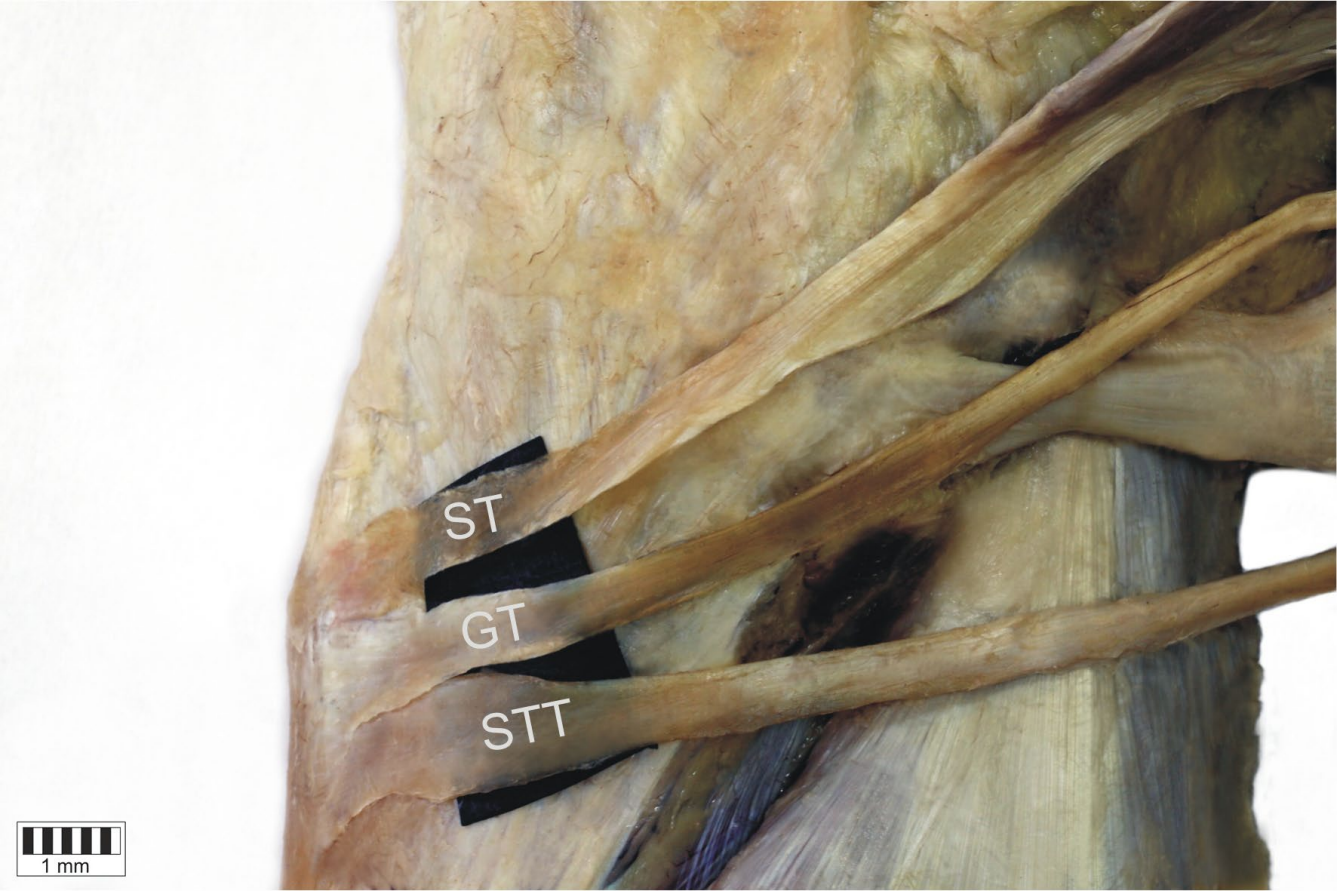
Type 1-1-1 insertion of the pes anserinus. Medial view of the right lower limb. *ST* sartorius tendon, *GT* gracilis tendon, *STT* semitendinosus tendon

**Fig. 3:**
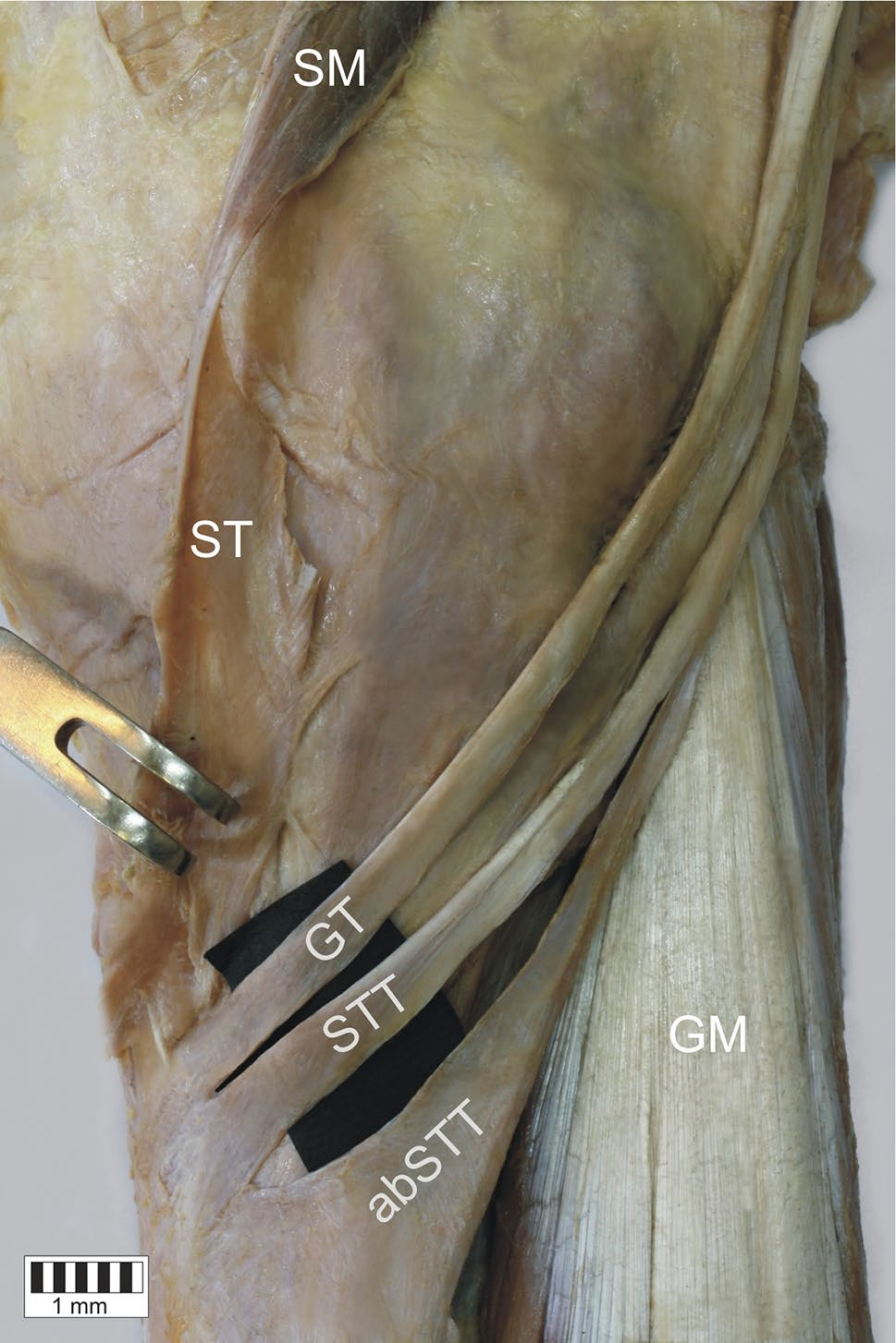
Type 1-1-2 insertion of the pes anserinus. Medial view of the right lower limb. *SM* sartorius muscle, *ST* sartorius tendon, *GT* gracilis tendon, *STT* semitendinosus tendon, *aSTT* accessory band of the semitendinosus tendon, *GM* gastrocnemius muscle

**Fig. 4:**
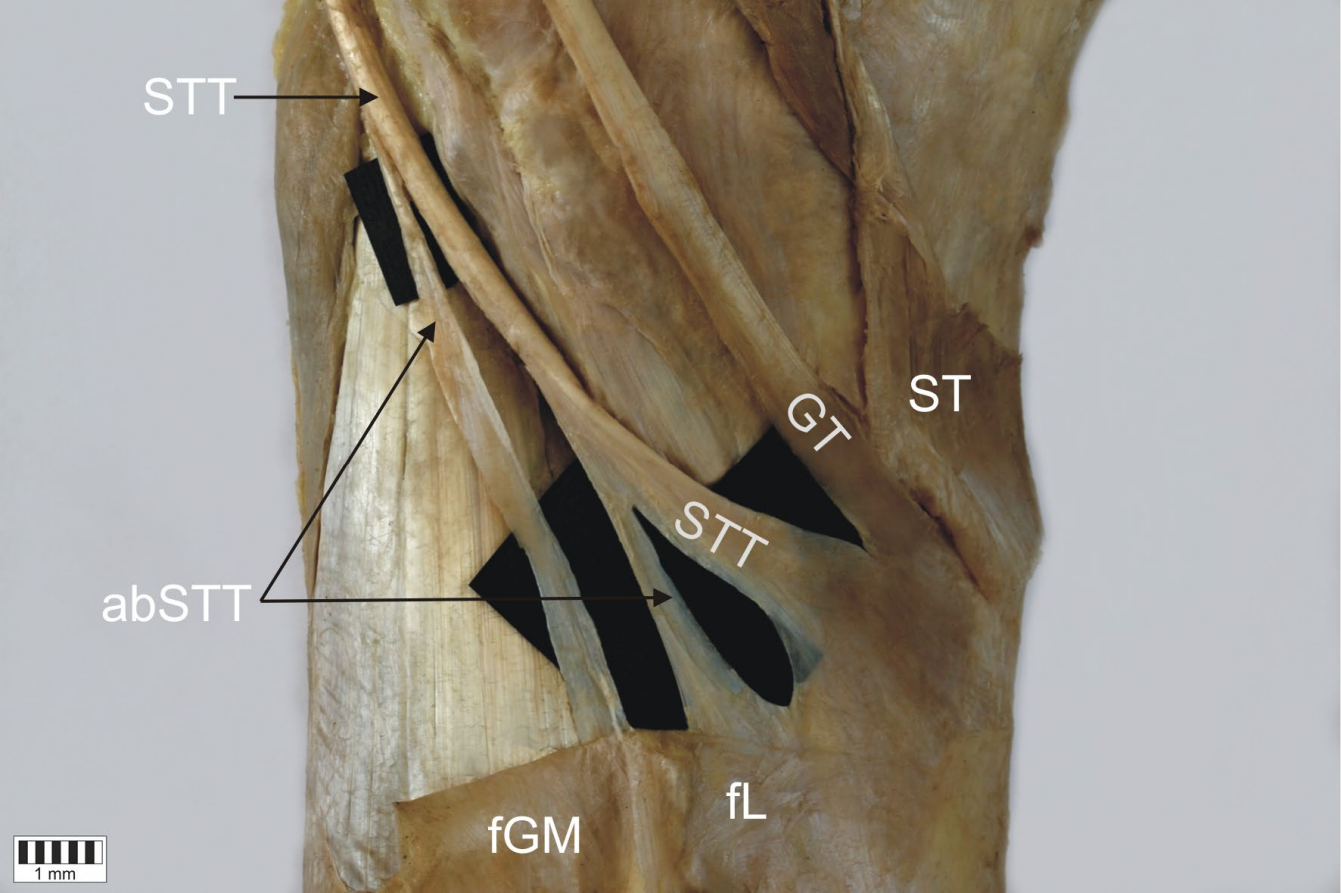
Type 1-1-3 insertion of the pes anserinus. Medial view of the left lower limb. *ST* sartorius tendon, *GT* gracilis tendon, *STT* semitendinosus tendon, *aSTT* accessory band of the semitendinosus tendon, *fGM* fascia of the gastrocnemius muscle, *fL* fascia of the leg

**Fig. 5:**
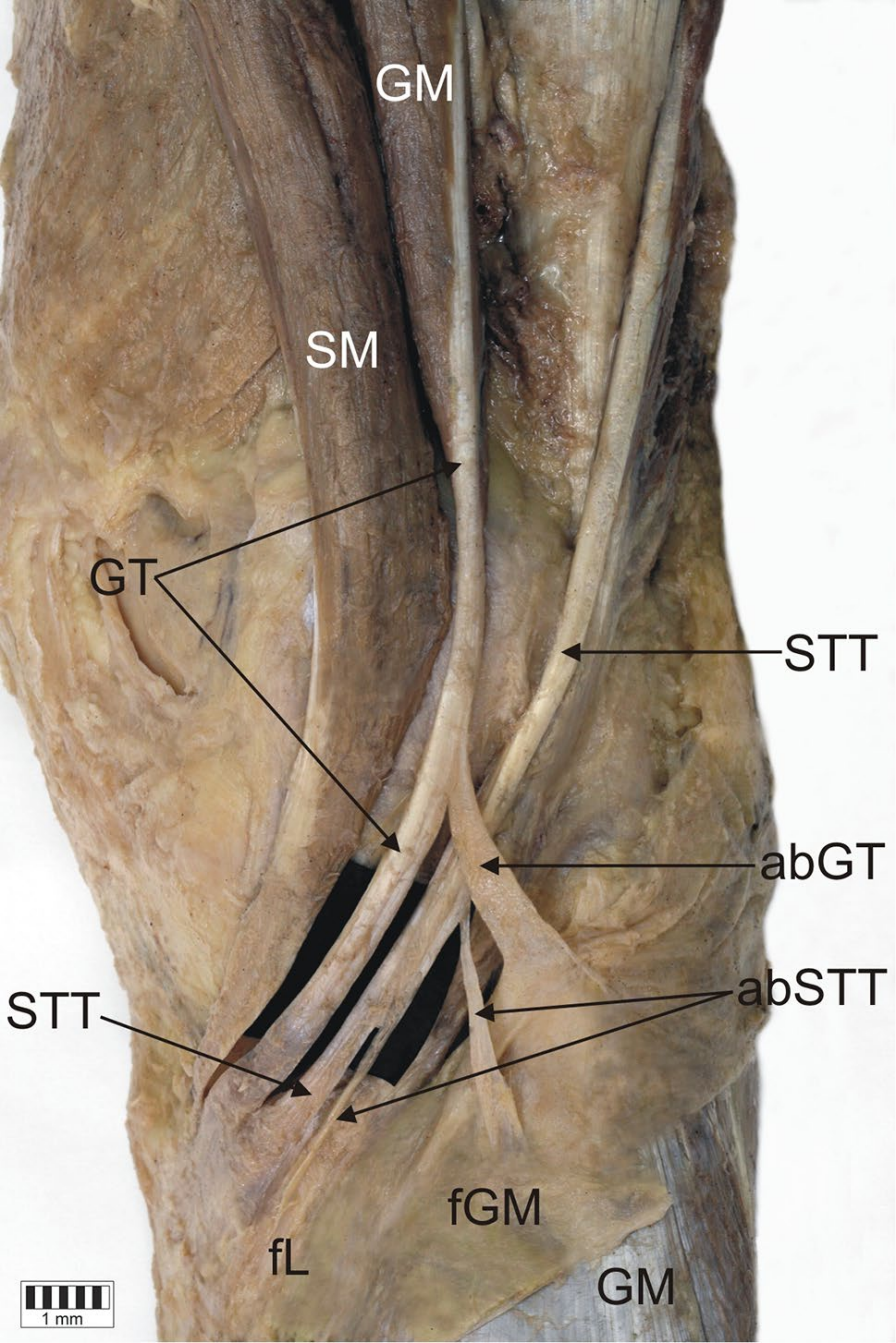
Type 1-2-3 insertion of the pes anserinus. Medial view of the right lower limb. *SM* sartorius muscle, *ST* sartorius tendon, *GT* gracilis tendon, *STTM* semitendinosus muscle, *STT* semitendinosus tendon, *aGT* accessory band of the gracilis tendon, *aSTT* accessory band of the semitendinosus tendon, *fL* fascia of the leg, *fGM* fascia of the gastrocnemius muscle, *GM* gastrocnemius muscle

**Fig. 6:**
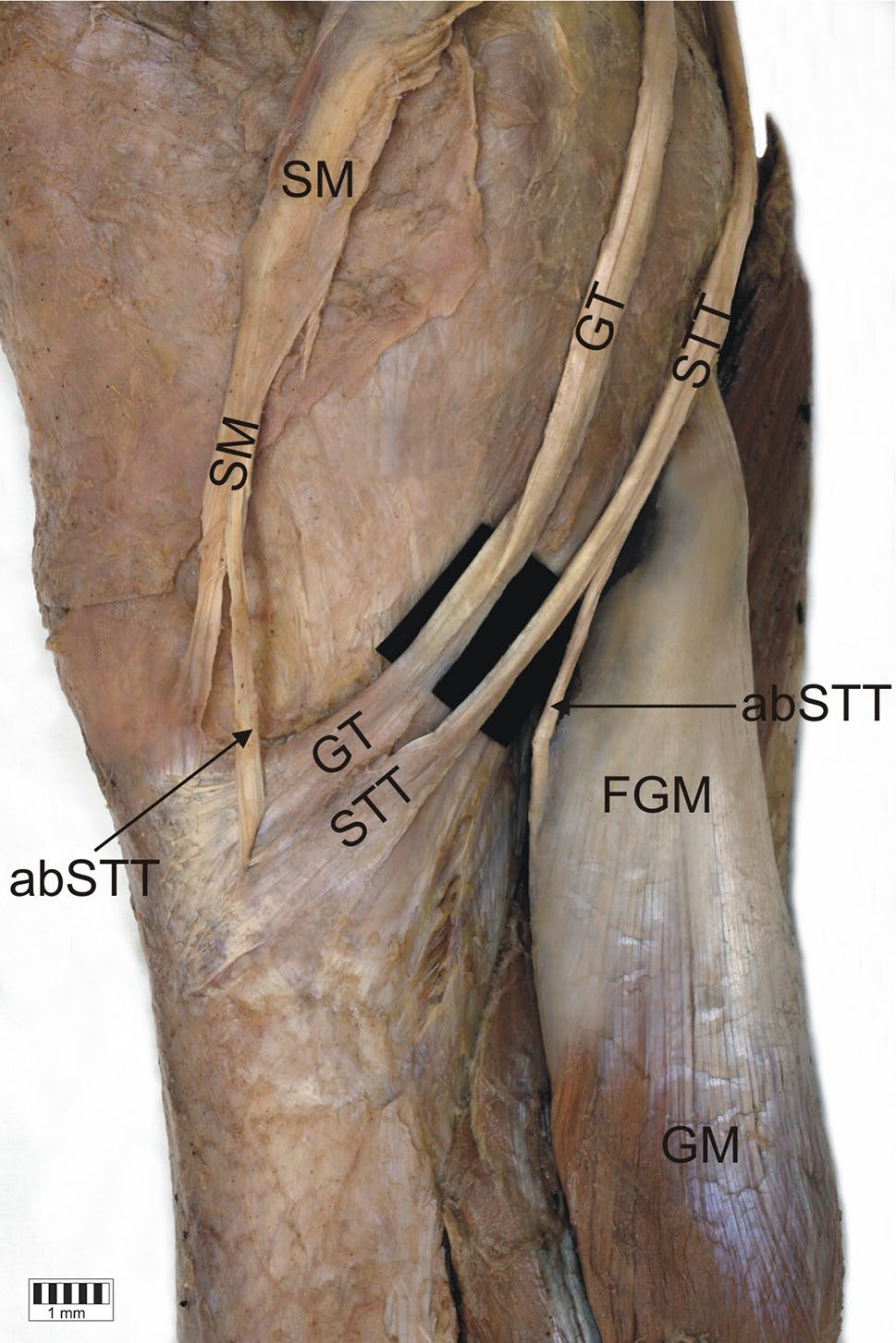
Type 2-1-2 insertion of the pes anserinus. Medial view of the right lower limb. *SM* sartorius muscle, *ST* sartorius tendon, *aST* accessory band of the sartorius tendon, *GT* gracilis tendon, *STT* semitendinosus tendon, *aSTT* accessory band of the semitendinosus tendon, *fGM* fascia of the gastrocnemius muscle, *GM* gastrocnemius muscle

**Fig. 7:**
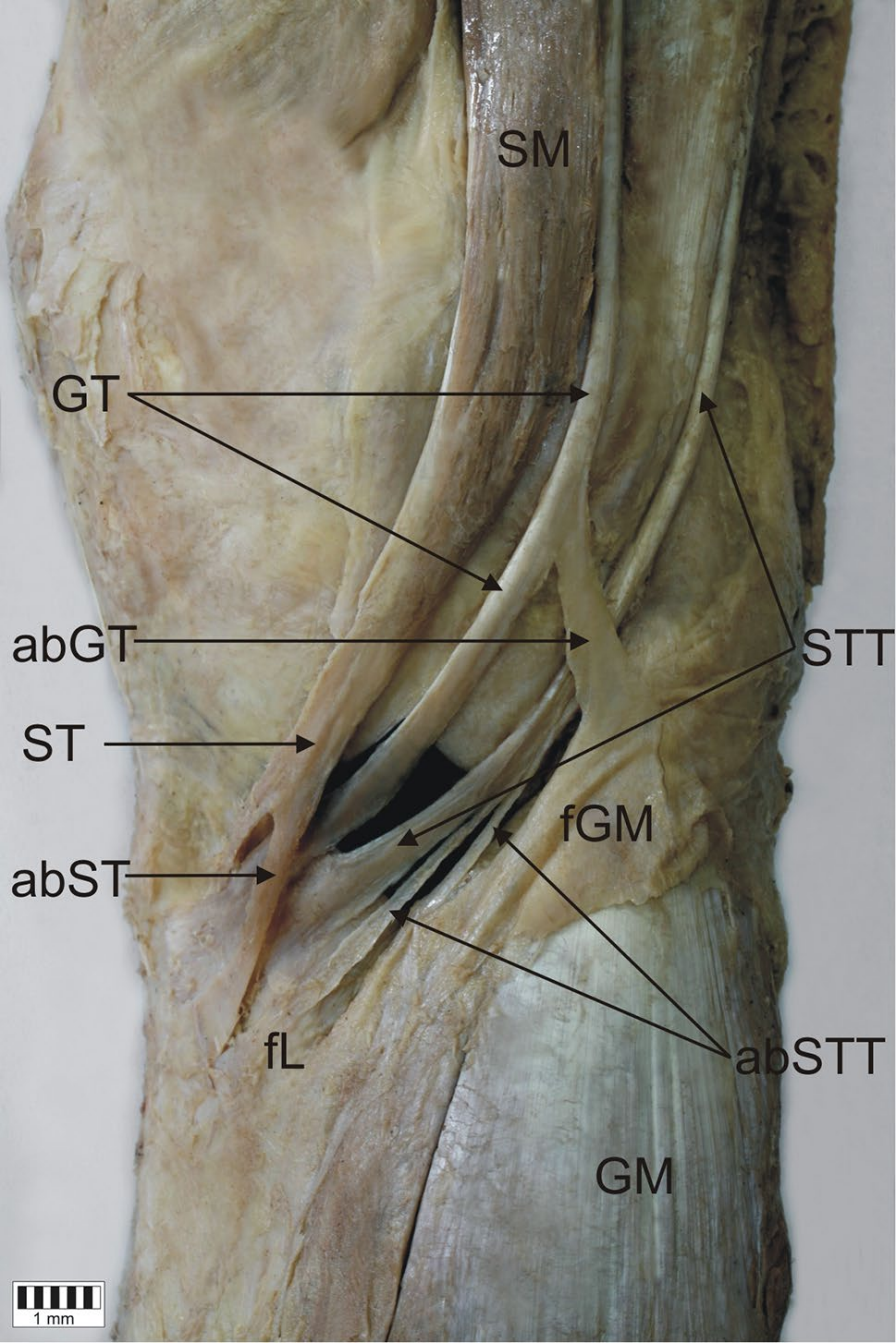
Type 2-2-3 insertion of the pes anserinus. Double insertion of the sartorius tendon. Medial view of the right lower limb. *SM* sartorius muscle, *ST* sartorius tendon, *abST* accessory band of the sartorius tendon, *GT* gracilis tendon, *abGT* accessory band of the gracilis tendon, *STT* semitendinosus tendon, *abSTT* accessory band of the semitendinosus tendon, *fGM* fascia of the gastrocnemius muscle, *GM* gastrocnemius muscle

Based on the distribution of tendons and accessory bands six types of PA were differentiated:

1. *Type 1-1-1* monotendinous ST, GT, STT. Present in 54 limbs (52.9%) (Fig. 2).
2. *Type 1-1-2* monotendinous ST and GT and one accessory band from STT. Present in 32 limbs (31.4%) (Fig. 3).
3. *Type 1-1-3* monotendinous ST and GT and two accessory bands from the STT. Present in nine limbs (8.8%) (Fig. 4).
4. *Type 1-2-3* monotendinous sartorius, one accessory band from the gracilis and two accessory bands from the semitendinosus. Present in one limb (1%) (Fig. 5).
5. *Type 2-1-2* one accessory band from sartorius, a single band from the gracilis and one accessory band from the semitendinosus. Present in two limbs (2%) (Fig. 6).
6. *Type 2-2-3* every muscle had one accessory band, one accessory band from sartorius and the gracilis, and two accessory band from the semitendinosus. Present in four limbs (3.9%) (Fig. 7).

### Type and location of insertion

**Fig. 8:**
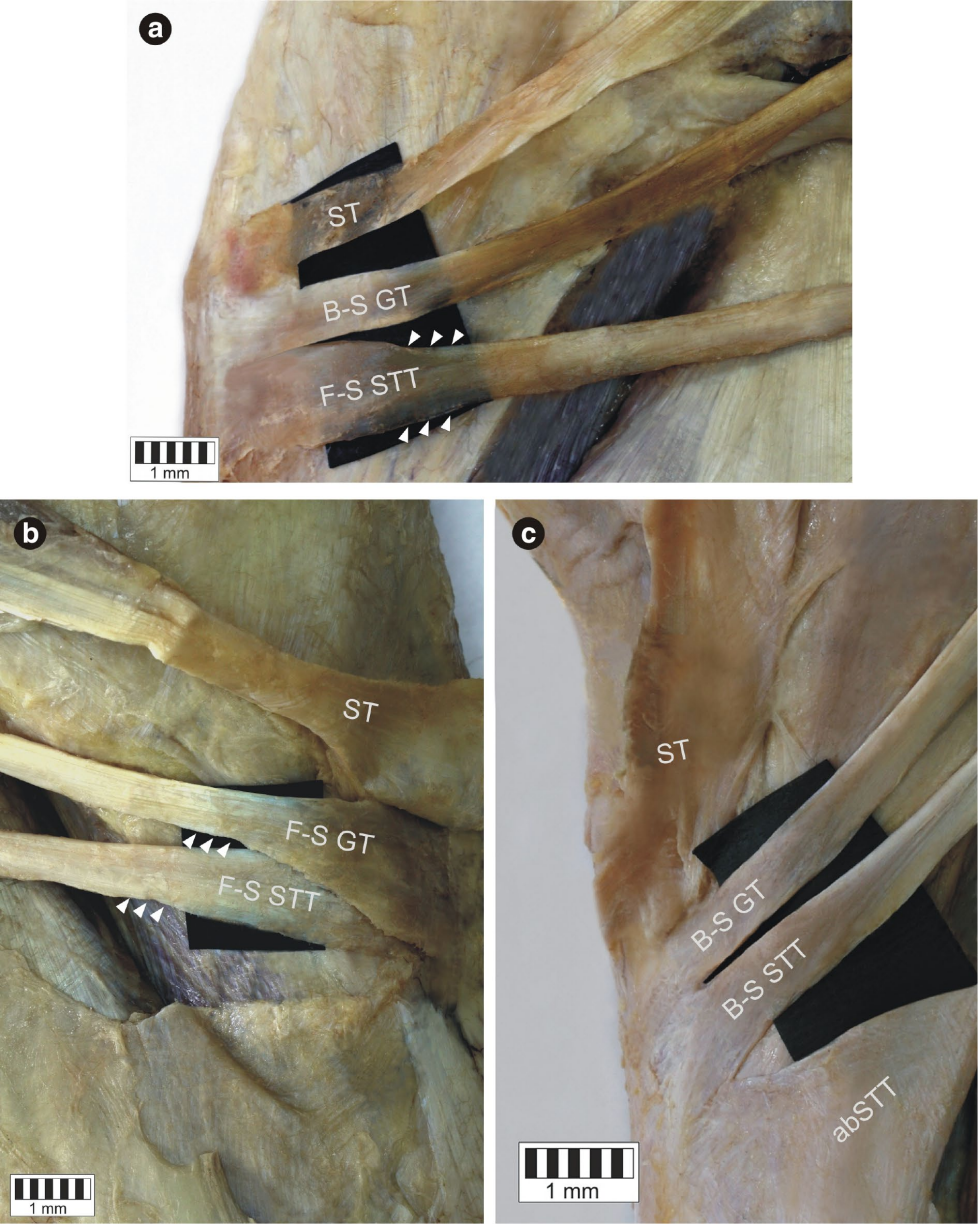
Types of the insertion of the gracilis tendon and semitendinosus tendon. **a** Band-shaped type gracilis tendon and fan-shaped type semitendinosus tendon. Medial view of the lower limb. *ST* sartorius tendon, *B-S GT* band-shaped type gracilis tendon, *F-S STT* fan-shaped type semitendinosus tendon. **b** Fan-shaped type gracilis and semitendinosus tendon. Medial view of the left lower limb. *ST* sartorius tendon, *F-S GT* fan-shaped type gracilis tendon, *F-S STT* fan-shaped type semitendinosus tendon. **c** Band shaped type gracilis and semitendinosus tendon. Medial view of the right lower limb. *ST* sartorius tendon, *B-S GT* band-shaped type gracilis tendon, *B-S STT* band-shape type semitendinosus tendon

Three types of insertion could be distinguished (Fig. 8a–c).

7. *Short* a short tendinous extension of the elongated muscle belly.
8. *Band-shaped* typified by an insertion less than two times wide than the tendon above.
9. *Fan-shaped* the insertion is at least twice the width of the tendon above.

**Fig. 9:**
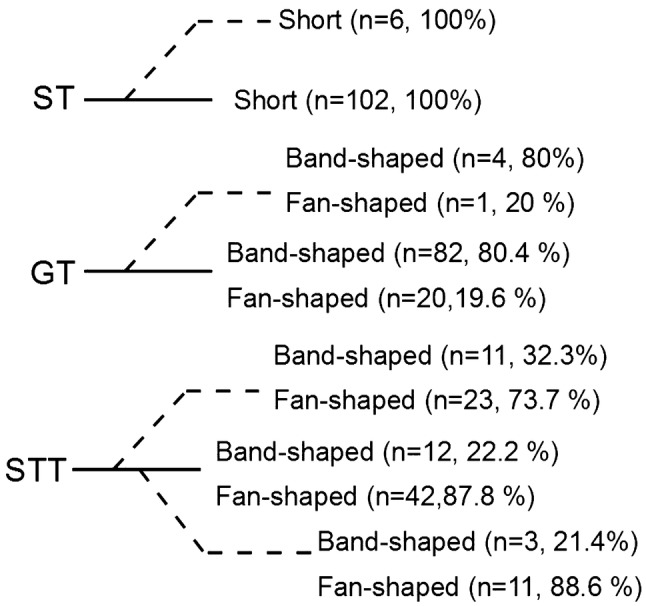
Types of insertion of the main band (continuous line) and additional bands (dashed line) for sartorius (ST), gracilis (GT) and semitendinosus (STT)

Distribution of types of tendons insertion according to the type of band are presented in Fig. 9.

### Morphometric measurements

Morphometric measurements did not differ between sexes (Supplementary Table 1). The findings regarding the accessory bands are as below.

#### Gracilis tendon

The point at which the accessory band departed from the GT was 29.8 mm below the muscle belly for the fan-shaped type and 31.8 mm for the band-shaped type. The length of the first accessory band was 22.0 mm in the fan-shaped type and 25.8 mm in the band-shaped type. These parameters were not significantly different (Supplementary Table 2).

#### Semitendinosus tendon

The first accessory band emerged from the muscle belly at a mean distance of 82.4 mm for the fan-shaped type, and 99.1 mm for the band-shaped type. The mean length of the first accessory band was 39.4 mm in the fan-shaped type, and 34.1 mm in the band-shaped type.

The second accessory band emerged from the muscle belly at 107.9 mm in the fan-shaped type, and 119.5 mm in the band-shaped type; its length was 33.1 mm for the fan-shaped type, and 22.1 mm for the band-shaped type. These parameters were not significantly different (Supplementary Table 2).

The mean length of the semitendinosus tendon between the insertion and the origin of the accessory band to the gastrocnemius muscle was 63.5 mm: 70.1 mm in the fan-shaped type and 49.9 mm in the band-shaped type (Supplementary Table 2).

Detailed information on the morphology of the insertion of the gracilis and semitendinosus muscles is presented in Table 1. Data presenting the localization of the insertion with regard to tibial tuberosity type is presented in Table 2.

**Table 1:** Parameters between the gracilis and the semitendinosus muscle

Parameter (mm)	Gracilis	Semitendinosus	*p* value
Width in distal attachment	8.42 (3.88)	11.23 (3.56)	0.0000
ExP thickness	2.29 (0.57)	1.91 (0.61)	(n.s.)
ExP width	7.46 (1.64)	7.75 (2.10)	(n.s.)
Distance between insertion and beginning of the ExP	28.42 (5.49)	27.88 (6.41)	(n.s.)
Muscle belly length	287.10 (36.55)	275.82 (39.59)	(n.s.)
Tendon length	139.57 (20.91)	152.11 (24.58)	0.0001
Width of tendon in myotendinous junction	5.13 (1.20)	6.17 (1.60)	0.0000
Thickness of tendon in myotendinous junction	5.59 (28.55)	2.96 (0.74)	(n.s.)
Width of tendon at the level of the 1st band	4.52 (0.53)	5.34 (1.19)	(n.s.)
Thickness of tendon at the level of the 1st band	2.47 (0.76)	2.41 (0.64)	(n.s.)
Width of the 1st band	2.89 (1.40)	2.41 (0.97)	(n.s.)
Thickness of the 1st band	1.04 (0.54)	1.01 (0.46)	(n.s.)
Width of tendon at the level of the 2nd band		4.58 (1.38)	
Thickness of tendon at the level of the 2nd band		1.94 (0.55)	
Width of the 2nd band		1.65 (0.86)	
Thickness of the 2nd band		0.66 (0.45)	

According to the Bonferroni correction, *p* < 0.004 is significant

**Table 2:** The distance between the insertion of the pes anserinus and tibial tuberosity (TT) (mm)

	Gracilis	Semitendinosus	*p* value
Medial to TT	2.66 (1.15)	3.21 (3.30)	0.0000
Inferior to TT	2.32 (9.02)	1.40 (3.82)	0.0000

The insertion was significantly wider for the semitendinosus tendon than the gracilis tendon (*p* = 0.0000). For the fan-shaped type insertion, the thickness of the tendon at the extension point was significantly greater in the gracilis muscle than the semitendinosus muscle (*p* = 0.0144). At the myotendinous junction, the semitendinosus tendon was significantly wider than the gracilis tendon (*p* = 0.0000).

## Discussion

The most important input of the present work is the systematic classification of the PA accessory bands and their insertion type.

Relatively little is known about the accessory bands of hamstring tendons. In addition, no classification of the PA has been drawn up, which would assist the planning of surgical procedures in this area. Previous anatomical studies on cadavers described only their variation with regard to the occurrence of accessory bands of the semitendinosus muscle [2, 9, 14, 16]. Alternatively, latest studies concerned mainly variation in the course and branching pattern of the infrapatellar branch of the saphenous nerve [17]. However, in these studies, little attention was paid to variants of PA tendons. LaPrade et al. [5] observed that the ST inserted to the superficial fascial layer, whereas the GT and STT are located in the deep surface of the superficial fascial layer. Similar results were obtained by Lee et al. [6], although they found the ST to be inserted deeper than the GT and STT in one case, and that the ST was bifurcated in another [6]. In the present study, no cases were observed where the ST was inserted deeper than the GT or STT; however, the ST was found to be bifurcated in six lower limbs. Candal-Couto et al. [2] reported that the accessory bands may arise from the GT or STT and insert separately into the PA. Accessory bands were also observed in the GT and STT. The GT had one–four accessory bands (usually two), while the ST had two–three accessory bands (usually two). Based on arthroscopies of 25 patients Yasin et al. [16] found the number of accessory bands to range from one to three in the GT, and from one to four in the STT. In addition, accessory bands were lacking only in one GT l, which was far more than what was observed in the current study. In a study of PA anatomy in 30 fresh cadaveric knees, the GT was found to have two accessory bands in five cases, and three accessory bands in only one case; in addition, the STT was found to have two accessory bands in seven cases but three accessory bands in only one case [14]. In a study of a Korean population [6], morphological variability was only observed for the STT, where 57 sampled lower limbs (66%) had one accessory band, 28 limbs (31%) had two and one limb (3%) had three.

A threefold classification was proposed by Ashaolu et al. [1] based on tendinosus formation, union of the tendons and point of insertion. Six types of tendinous formation were proposed (type I–VI), with the most common type being monotendinous ST, GT and STT with one accessory band. A comparison of these results with our own is presented in Table 3. The number of bands originating from the GT and STT is an important taxonomic category; however, due to the great variability inherent in PA morphology, it is likely that the numbers of specimens used in previous studies were too low to reflect all the important variations [2, 14, 16]. Although the classification based on tendinous formation proposed by Ashoulu et al. [1] focuses on detailed anatomical aspects, a classification proposed in this research may be more clinically relevant to facilitate more effective tendon grafting. Unlike other studies, our findings indicate that the most common type of PA comprised the monotendinous ST, GT and STT, accounting for 54 out of 102 cases (52.9%).

**Table 3:** Comparison of classification

Classes	Ashaolu et al. [1]	Current study
ST + GT + STT	1 (5%)	54 (52.9%)
ST + GT + STT + aSTT + SMT + TCL	1 (5%)	0 (0%)
ST + GT + STT + aSTT	13 (65%)	32 (31.4%)
ST + GT + STT + aSTT + SMT	3 (15%)	0 (0%)
ST + GT + STT + aST + SMT	1 (5%)	0 (0%)
ST + GT + STT + aGT + aSTT	1 (5%)	0 (0%)
ST + GT + aSTT + aSTT	0 (0%)	9 (8.8%)
ST + GT + STT + aGT + aSTT + aSTT	0 (0%)	1 (1%)
ST + GT + STT + aST + aSTT	0 (0%)	2 (2%)
ST + GT + STT + aST + aGT + aSTT + aSTT	0 (0%)	4 (3.9%)

*ST* sartorius tendon, *GT* gracilis tendon, *STT* semitendinosus tendon, *SMT* semimembranosus tendon, *TCL* tibial collateral ligament, *aSTT* accessory band of semitendinosus tendon, *aGT* accessory band of the gracilis tendon, *aST* accessory band of the sartorius tendon

The type of insertion is a second feature in our classification. For the GT and STT a band-shaped and a fan-shaped types of insertion were recognised. The fan-shaped form occurred in 20 cases (19.6%) for the GT and in 82 cases (80.4%) for the STT. In addition, it can be present in both the main tendon and the additional bands. This might be an important feature because, for the fan-shaped tendon, it might be harder to recognize the correct tendon and to expose the point where the tendon stripper can be placed. Interestingly, earlier studies have failed to identify the type of insertion of the PA tendon [1, 2, 6, 9, 14, 16, 17].

Last but not least, to identify the correct tendons for grafting, the place of insertion should be recognised. During a surgical procedure, concrete landmarks are required to navigate the site of incision and to identify tendons. As the tibial tuberosity is arguably the best candidate for this purpose, lines A and B (Fig. 1) were proposed as measurements to better locate these tendons. Although this is helpful for localizing the main tendons of the gracilis and semitendinous, accessory bands might be a potential cause of complications [3, 7, 13, 15].

There are two important aspects for recognizing accessory bands—their length and point of insertion. It is believed that inspection of 10 cm proximal to the insertion of PA ensures the safety in presence of accessory bands. However, Candal-Cauto et al. [2] reported the origin of accessory bands more than 10 cm proximal from PA insertion in eight of ten tested specimens. Tuncay et al. [15] noted that accessory bands started 6–8 cm from insertion and ended within 8–12 cm. Nevertheless, in the present study, the origin of the accessory bands was localized 63.5 mm from the main PA insertion and the mean length of the accessory band was 63.5 mm for the STT (70.1 mm in fan-shaped and 49.4 mm in band-shaped types) and 118.8 mm for the GT (117.7 mm in fan-shaped and 119.3 mm in band-shaped types). Thus, our study suggests that a margin of 10 cm is sufficient.

The location of the insertion of accessory bands is not fully known. Candal-Cauto et al. [2] observed accessory bands in 20 lower limbs (100%), whereas Ashaolu et al. [1] observed them in 19 lower limbs (95%); however, in the present study, they were only observed in 47.1%. The accessory bands of the STT were reported to insert into the gastrocnemius fascia or popliteal fascia, while those of the GT into the sartorius or gastrocnemius muscle or deep fascia [2]. Reina et al. reported the presence of accessory bands within the GT, running towards the fascia of the medial head of the gastrocnemius muscle, in 17 of 29 studied cases [14], while the other band followed a different course. Generally speaking, in accordance with our findings, the accessory bands of the STT always ran towards the aponeurosis of the medial head of the gastrocnemius muscle. However, no insertion into the popliteal fascia was identified. Due to the restricted field of view through the small incision, inspection of the area 10 cm proximal to the PA insertion might be difficult; therefore, the best feature for identifying the presence of an accessory band would be to recognize the fusion with fascia.

The present study does have some limitations, one being that no sample size calculation was performed; however, the study is the largest cadaver-based study yet performed on the anatomy of the PA. Second, being heterogeneous, the proposed classification depends on several morphological details, such as type of insertion or presence of accessory bands. In addition, as the dissected lower limbs were obtained from different donors, it was not possible to evaluate the symmetry of the types. Finally, as this is only an anatomical study, a spectrum of variation could be presented. As the pattern of PA tendons and their accessory bands varies according to the individual, it would be desirable to be able to recognise the anatomy of this area in patients being prepared for surgery. Further studies should examine the potential value of ultrasound or MRI for this purpose. Nonetheless, this study helps raise awareness of “what and where” to look for, and offers a uniform classification and terminology to act as a foundation for communication with surgeons. Surgical harvesting of tendons can be facilitated by our findings. First, the proposed classification recognises the possibility of the existence of additional bands for each PA tendon and provides information on their topography and morphology. Second, knowledge of these variants and application of the proposed classification may serve as common practice for specialists if modification of the surgical techniques is introduced. Third, this work might provide a segue to studies on diagnostic imaging to determine PA morphological variations *in vivo* and not to mistake them with pathologies of the PA.

## Conclusion

Introducing a new classification may help improve the planning of surgical procedures, because every uncommon formation of PA increases the risk of failure while harvesting the tendons. Differently shaped tendons can affect the ease of tendon harvesting. The careful use of a tendon stripper 10 cm proximal to the PA insertion is essential. The morphological variability of the PA is considerable, particularly regarding the distance from the PA insertion of accessory bands and their shape, and the present work is the first to examine its nature in such great detail.

## Electronic supplementary material

Below is the link to the electronic supplementary material.

Supplementary material 1 (DOC 37 KB)
