# Supplementary material for: A proposal for a new classification of pes anserinus morphology
Source: Knee Surg Sports Traumatol Arthrosc. 2018 Dec 10;27(9):2984–93. doi: 10.1007/s00167-018-5318-3 (PMC6706366; doi:10.1007/s00167-018-5318-3)
Supplement: Supplementary file 1 — Supplementary material 1 (DOC 37 KB) [file 167_2018_5318_MOESM1_ESM.doc]

Table 1. Morphometric lower limbs measurements

| Parameter [mm] | Females (n=40) | Males (n=62) | p-value |
| --- | --- | --- | --- |
| Limb length | 775.0 (60.5) | 785.5 (62.6) | (n.s.) |
| Thigh length | 364.1 (28.2) | 371.3 (34.0) | (n.s.) |
| Crus length | 410.8 (34.9) | 412.6 (33.9) | (n.s.) |

**Table 2. Characteristics of tendons in relation to the branching of accessory bands.**

|  | Parameter [mm] | General | Fan-shaped | Band-shaped | p-value |
| --- | --- | --- | --- | --- | --- |
| Gracilis | Length of tendon above the accessory band [mm] | 31.10 (1.27) | 29.78 (11.32) | 31.77(14.31) | (n.s.) |
|  | Length of tendon below the accessory band [mm] | 118.78 (24.87) | 117.69 (39.12) | 119.32 (35.14) | (n.s.) |
|  | Length of the accessory band [mm] | 24.52 (6.79) | 22.04 (9.87) | 25.77 (9.11) | (n.s.) |
| Semitendinosus | Length of tendon above the first accessory band [mm] | 87.79 (24.29) | 82.44 (18.74) | 99.15 (31.66) | (n.s.) |
|  | Length of tendon below the first accessory band [mm] | 63.51 (29.08) | 70.15 (29.66) | 49.38 (23.53) | (n.s.) |
|  | Length of the first accessory band [mm] | 37.71 (14.64) | 39.40 (14.18) | 34.12 (15.92) | (n.s.) |
|  | Length of tendon above the second accessory band [mm] | 111.11 (24.12) | 107.94 (27.47) | 119.54 (11.05) | (n.s.) |
|  | Length of tendon below the accessory band [mm] | 52.12 (38.73) | 56.06 (13.20) | 41.63 (45.04) | (n.s.) |
|  | Length of the second accessory band [mm] | 30.13 (9.08) | 33.15 (8.75) | 22.06 (3.12) | (n.s.) |
